# Comparison of the Effect of Amaranth Oil vs. Rapeseed Oil on Selected Atherosclerosis Markers in Overweight and Obese Subjects: A Randomized Double-Blind Cross-Over Trial

**DOI:** 10.3390/ijerph18168540

**Published:** 2021-08-12

**Authors:** Małgorzata Jamka, Anna Morawska, Patrycja Krzyżanowska-Jankowska, Joanna Bajerska, Juliusz Przysławski, Jarosław Walkowiak, Aleksandra Lisowska

**Affiliations:** 1 Department of Pediatric Gastroenterology and Metabolic Diseases, Faculty of Medicine, Poznan University of Medical Sciences, Szpitalna Str. 27/33, 60-572 Poznań, Poland; mjamka@ump.edu.pl (M.J.); pkrzyzanowska@ump.edu.pl (P.K.-J.); jarwalk@ump.edu.pl (J.W.); 2 Department of Bromatology, Faculty of Pharmacy, Poznan University of Medical Sciences, Marcelińska Str. 42, 60-354 Poznań, Poland; akm@ump.edu.pl (A.M.); jprzysla@ump.edu.pl (J.P.); 3 Department of Human Nutrition and Dietetics, Faculty of Food Science and Nutrition, Poznan University of Life Sciences, Wojska Polskiego Str. 31, 60-624 Poznań, Poland; joanna.bajerska@up.poznan.pl; 4 Department of Clinical Auxology and Pediatric Nursing, Faculty of Health Sciences, Poznan University of Medical Sciences, Szpitalna Str. 27/33, 60-572 Poznań, Poland

**Keywords:** adiponectin, apolipoproteins, oxidized low-density lipoprotein, tumor necrosis factor-alpha

## Abstract

It is well known that rapeseed oil improves lipid profile and has antiatherosclerotic properties. Recently, amaranth oil has also become popular due to its potential health benefits. However, the effect of this oil on atherosclerosis markers in humans is not clear. Therefore, this study aimed to compare the effect of amaranth and rapeseed oils on selected atherosclerosis-related parameters in overweight and obese subjects. In this randomized cross-over study, 44 subjects were instructed to consume 20 mL of amaranth oil and rapeseed oil during two consecutive three-week intervention periods separated by a washout period of the same duration as the intervention. The outcome variables included changes in tumor necrosis factor-alpha, adiponectin, oxidized low-density lipoprotein, apolipoproteins (Apo) A1, B and E as well as glucose and insulin homeostasis markers. Compared to rapeseed oil, amaranth oil had a slight positive effect on adiponectin levels (mean (95% confidence interval): 0.55 (0.22–0.89) vs. -0.29 (−0.75–0.16), *p* = 0.0002) but negatively affected ApoB concentrations (0.05 (−0.01–0.11) vs. 0.03 (−0.07–0.00), *p* = 0.0004) and ApoB/A1 ratio (0.01 (−0.03–0.05) vs. −0.02 (−0.04–0.00), *p* = 0.0113). No differences between the other analyzed parameters were observed. In conclusion, amaranth oil does not have a greater beneficial effect on atherosclerosis markers than rapeseed oil. However, further studies with a longer intervention period are needed. The study was retrospectively registered with the German Clinical Trials Register within the number: DRKS00014046, date of registration: 3 May 2018.

## 1. Introduction

Atherosclerosis is one of the most important causes of global mortality [1], while obesity is a well-known risk factor for the development of atherosclerosis [2], with both conditions associated with low-grade inflammation and increased levels of oxidative stress markers. Previous studies have also shown increased secretion of apolipoprotein (Apo) B, several cytokines and oxidized low-density lipoprotein (ox-LDL) in addition to reduced ApoA1, ApoE and adiponectin levels in subjects with obesity and atherosclerosis [3]. Therefore, a new dietary strategy that could help to manage these parameters is needed.

Canola belongs to the *Brassicaceae* family, which includes the following species: *juncea, napus* and *rapa*. Brassica *napus* and *rapa* are commonly known as rapeseed [4]. Over the last decades, canola has become one of the most popular oilseeds in the world [5]. Canola (or rapeseed) oil is characterized by low levels of saturated fatty acids (SFAs) and high amounts of unsaturated fatty acids (UFAs; e.g., oleic acid, linoleic acid and alpha-linolenic acid) [6]. The oil also contains plant sterols and tocopherols [7,8] and is known for cardio-protective activities through antithrombic, antioxidative and lipid-lowering effects [9]. Besides, it has been suggested that a high amount of mono-unsaturated fatty acid (MUFA) in canola oil may protect low-density lipoprotein cholesterol (LDL-C) from oxidation [9,10]. Additionally, canola oil consumption was associated with immunomodulatory activities [9,11].

Amaranth (*Amaranthus L.)* belongs to the pseudocereal group and is grown in Asia and the Americas [12,13]. Among the many species of amaranth, the most popular are *Amaranthus caudatus L*., *Amaranthus cruentus L.* and *Amaranthus hypochondriacus L*. [14]. Amaranth is cultivated mostly for its seeds, from which flour and oil are produced [12]. The quantity of oils in amaranth seeds is higher than most other grains. It has been shown that amaranth grain generally consists of around 6–7% oil depending on the species and the genotype. Importantly, amaranth oil contains approximately 75% UFA and is high in linoleic, oleic and α-linoleic acid, with n-6 to n-3 polyunsaturated fatty acid (PUFA) ratio of 44.34:1. However, amaranth oil also contains SFA, e.g., palmitic and stearic acid, and the ratio of SFA to UFA is approximately 1:3 [15]. Amaranth oil is also unique due to the high content of bioactive substances, including squalene, tocopherols, sterols and others [16].

Recently, amaranth has become popular due to its potential medicinal properties. Several studies investigated the hypolipemic effect of intervention with amaranth in animals [17,18] and humans [19,20,21]. Studies in animal models demonstrated the ability of amaranth to decrease total cholesterol (TC) and LDL-C levels [17,18]. However, studies in humans were not convincing regarding amaranth’s lipid-lowering activity [19,20,21]. Moreover, the effect on high-density lipoprotein cholesterol (HDL-C) remains unclear [22,23] as well as the components that are potentially responsible for its favorable effect. Some studies showed that squalene can have a hypocholesterolemic effect, while other studies suggested that other components of amaranth are responsible for the effect [20,24]. Notwithstanding, in vitro studies also showed the potential of amaranth to influence hypertension [25,26,27]. Martirosyan et al. [20] also showed that amaranth oil combined with an antiatherogenic diet decreases systolic blood pressure. The antidiabetic activity of amaranth oil was also demonstrated in animals [28] and humans [29]. Previously, Gonor et al. [30] also reported the positive effect of amaranth oil supplementation on antioxidant and immune status in subjects with ischemic heart disease and hyperlipoproteinemia. Some studies also found a beneficial effect of amaranth on anthropometric parameters [29,31], but other studies did not confirm these findings [19]. The effect of amaranth oil supplementation on early atherosclerosis parameters (e.g., Apos and ox-LDL levels) in humans is also not clear, as most previous studies regarding such parameters were performed in animal models [18,32], but the metabolic pathways in animals differ from those in humans, so it is not easy to extrapolate these results to humans.

Therefore, this study aimed to compare the effect of amaranth and rapeseed oils on selected atherosclerosis markers in overweight and obese subjects. Previously, we demonstrated that amaranth oil significantly increased TC and LDL-C levels compared to rapeseed oil. However, no significant differences between groups were observed for anthropometric parameters, HDL-C, triglycerides, atherogenic index of plasma, C-reactive protein (CRP), asymmetric dimethylarginine, vascular adhesion molecule-1 and soluble *p*-selectin [19]. Taking into account the negative effect of amaranth oil on lipid profile, we decided to extend the analyses to other atherogenic markers for which the effect of the amaranth oil were not previously assessed in humans (tumor necrosis factor-alpha (TNF-α), adiponectin, ox-LDL, ApoA1, ApoB and ApoE). Besides, glucose, insulin levels, homeostatic model assessment for insulin resistance (HOMA-IR) and quantitative insulin sensitivity check index (QUICKI) were also measured.

## 2. Materials and Methods

The study was designed as a randomized double-blind cross-over trial per the standards of CONSORT (see Supplementary Materials, Table S1) [33] and conducted according to the guidelines in the Declaration of Helsinki. The study protocol was approved by the Poznan University of Medical Sciences Bioethical Committee (refs. 359/14 and 870/14) and registered in the German Clinical Trials Register database under the number DRKS00014046 (date of registration: 3 May 2018). All participants received information about the study and that participation was voluntary. All subjects were aware that they could withdraw at any time without providing reasons.

Overweight (body mass index (BMI): 25–29.9 kg/m^2^) and obese (BMI ≥ 30 kg/m^2^) adults were recruited. The exclusion criteria included a history of chronic systemic or gastrointestinal diseases, hepatic disease, exocrine pancreatic insufficiency, conjugated linoleic acid supplementation during the study and within a month before starting the study, treatment with statins, other drugs or dietary supplement affecting fat digestion or absorption (e.g., orlistat, chitosan, green tea), as well as pregnancy and breastfeeding. Potential subjects were recruited via written advertisements distributed in Poznań. After telephone contact, the potential subjects were screened by a physician during an inclusion visit to comply with protocol requirements.

The study intervention has been described in detail previously [19]. Briefly, all participants were randomly assigned (allocation ratio: 1:1) to arms I and II. In arm I, amaranth oil was administered in a dose of 20 mL per day in the first intervention, and rapeseed oil in a dose of 20 mL per day was provided in the second intervention. All subjects received the same dose of oils. In arm II, subjects received rapeseed oil in the first intervention and amaranth oil in the second intervention. The dose of both oils in arm II was the same as in arm I. The duration of both interventions was three weeks and they were separated by a three-week washout period. The amaranth oil used in the study was extracted from *Amaranthus cruentus L*. and produced by the Szarłat company (Łomża, Poland). The rapeseed oil was extracted from *Brassica napus L.* and was supplied by the Vitacorn company (Poznań, Poland). Both oils were cold-pressed and stored in identical dark bottles. According to the manufacturer’s instructions, both oils were kept in refrigerated storage. During the intervention, participants were instructed to not change their dietary habits and physical activity. Importantly, the doses of applied oils were exchanged for the same amount of fat used in the diet. Therefore, the energy contents of the diet did not change during the intervention. Moreover, during the intervention, subjects were supervised over telephone by the nutritionist to check compliance with the study protocol, especially regular administration of the appropriate oil. To verify adherence to the study protocol, participants were instructed to regularly return empty oil bottles to the research team. No deviation from the study protocol was noted.

The primary outcomes of the study were the comparison of the effect of amaranth and rapeseed oils on CRP levels. Here, we reported the results for secondary outcomes, such as TNF-α, adiponectin, ox-LDL, ApoA1, ApoB and ApoE, as well as glucose and insulin homeostasis markers. All outcomes measurements were collected at the Poznan University of Medical Sciences before and after each intervention period. Most of the analyzed parameters were assessed at the Poznan University of Medical Sciences, except glucose and insulin levels, which were analyzed in the ALAB Laboratory (Poznań, Poland). All outcomes were assessed by the same methods in both arms.

At baseline, anthropometric parameters, including body weight and body height, were measured to calculate BMI to check if participants fulfilled the overweight or obesity criteria. All measurements were performed fasting and during the measurements, participants were dressed in light clothing and were barefoot.

Pre- and post-intervention fasting blood samples were collected using standard methods and stored at −70 °C until analysis. The following parameters were measured using the enzyme-linked immunosorbent assay methods: TNF-α (DRG Instruments GmbH, Marburg, Germany), adiponectin (Mediagnost, Reutlingen, Germany), ox-LDL (Shanghai Sunred Biological Technology, Shanghai, China) and ApoE (Assay Max Human Lipoprotein E, Assaypro, St. Charles, MO, USA). ApoA1 and ApoB levels were assessed by an immunonephelometric method (Siemens Healthcare Diagnostics Products GmbH, Marburg, Germany). Glucose levels were assessed by the enzymatic method with hexokinase, while insulin levels were analyzed using the electrochemiluminescence method. HOMA-IR [34] and QUICKI [35] ratios were also calculated.

Block randomization was done by an independent researcher using computer software (Excel, Microsoft Corp, Redmond, WA, USA). A randomization list was generated and the allocation sequence was concealed until assignment to interventions. Neither the study participants nor the research staff were aware of the allocation sequences.

The minimum sample size was calculated using the G*Power software (University of Kiel, Kiel, Germany) based on the following assumptions: the probability of a type I error at an α = 0.05; the probability of a type II error at a β = 0.2; the means differences equal to 20%; standard deviation (SD) equal to 30% of the mean; and the allocation ratio: 1:1. Based on the assumption, the minimum sample size was 35 subjects. Anticipating a maximum 20% dropout rate, at least 44 subjects should be recruited for such a study. The calculation was performed based on changes in our primary outcome. However, we also retrospectively calculated a minimum sample size based on changes in HOMA-IR reported previously by Moszak et al. [29]. The calculation showed that at least 12 participants should be recruited for the study.

The Statistica 13 software (TIBCO Software Inc., Palo Alto, CA, USA) was used to perform the statistical analysis and a *p*-value < 0.05 was considered significant. The data distribution was checked using the Shapiro–Wilk (normality) test. Data were presented as mean and SD with 95% of confidence interval (95%CI) as well as the median and interquartile range (IQR). As most of the data were not normally distributed, the Wilcoxon test was used to compare the effect of amaranth and rapeseed oils on analyzed parameters. Differences between arm I and arm II were assessed using the Mann–Whitney U test. The Chi^2^ test was used to compare the categorical variables.

## 3. Results

**Figure 1:**
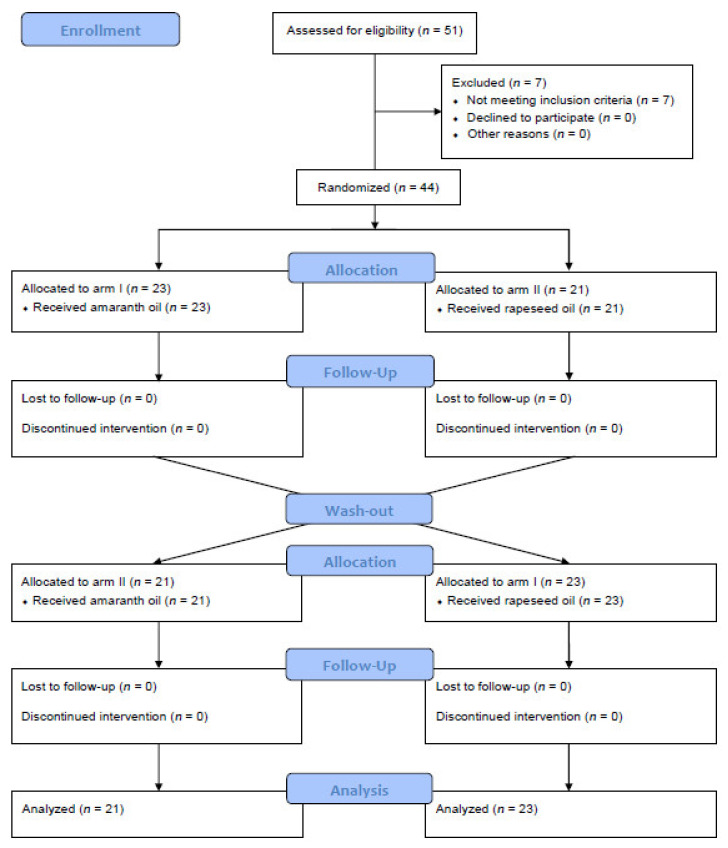
The CONSORT 2010 flow diagram.

The recruitment process began in February 2015 and finished in August 2017, while the intervention period finished in October 2017. The participant flowchart is presented in Figure 1. Among 51 subjects who were assessed for eligibility, seven did not meet the inclusion criteria and were excluded from the study. In total, 44 participants were randomized into arm I (*n* = 23) and arm II (*n* = 21), received the allocated intervention and completed the study. No serious side effects were noted.

Baseline characteristics of the study population are presented in Table 1, with no differences between subjects allocated to arm I and arm II observed.

The differences (Δ, post- minus preintervention values) between the effect of amaranth and rapeseed oils on analyzed markers are presented in Table 2. Both types of intervention significantly differed in the effect on adiponectin (amaranth oil vs. rapeseed oil (mean (95% CI): 0.55 (0.22–0.89) vs. −0.29 (−0.75–0.16), *p* = 0.0002) and ApoB levels (0.05 (−0.01–0.11) vs. 0.03 (−0.07–0.00), *p* = 0.0004) as well as ApoB/A1 ratio (0.01 (−0.03–0.05) vs. −0.02 (−0.04–0.00), *p* = 0.0113) but with no differences observed for the other analyzed parameters.

## 4. Discussion

Previously, we reported that, in comparison to rapeseed oil, amaranth oil significantly increased TC and LDL-C concentrations [19]. Based on the obtained results, we extended the analyses to other atherogenic parameters and demonstrated that amaranth oil may have a beneficial effect on adiponectin levels compared to rapeseed oil but simultaneously slightly increased ApoB concentrations, consequently the ApoB/A1 ratio. No differences between the effect of amaranth oil and rapeseed oil on glucose and insulin homeostasis markers, ApoA1, ApoE, ox-LDL and TNF-α levels were observed.

It is well known that obesity promotes chronic low-grade inflammation, which may be associated with the development of atherosclerosis and cardiovascular diseases. The most important pro-inflammatory parameters, elevated levels of which are frequently observed in chronic low-grade systemic inflammation, are interleukin (IL) 1β, IL-6, TNF-α and CRP [36]. Previously, it has been suggested that amaranth may stimulate the immune system, thus decrease pro-inflammatory cytokine levels. However, the data assessing the effect of amaranth oil on inflammatory markers is limited. Gonor et al. [30] investigated the effect of amaranth oil supplementation on immune status in subjects with ischemic heart disease and hyperlipoproteinemia, demonstrating that a combination of an antiatherosclerotic diet with 600 mg of squalene in the form of amaranth oil has a positive effect on immune status. However, no differences between the effect of amaranth and rapeseed oils on TNF-α levels were found in the present study. These results are in line with our previous findings, which showed no differences between the effect of both types of oils on CRP levels [19].

Important parameters that may be associated with the atherosclerosis process are Apos and ox-LDL levels. ApoA is the main protein of HDL-C, while ApoB is the main component of LDL-C. ApoA1 concentrations are inversely and ApoB levels are positively correlated with the risk of cardiovascular diseases. Therefore, it has been suggested that the ApoB/ApoA1 ratio predicts cardiovascular risk more accurately than lipid profile. ApoE also plays an important role in lipid metabolism and is associated with very-low-density lipoproteins, intermediate-density lipoproteins, chylomicron remnants, and certain subclasses of HDL-C. Higher levels of ApoE are a risk factor for cardiovascular diseases [37]. ox-LDL may play a crucial role in atherosclerosis initiation and progression. The proatherogenic role of ox-LDL is associated with its chemotactic and proliferating actions on monocytes and macrophages, stimulation of smooth muscle cell recruitment, and proliferation in the tunica intima and induced apoptosis [38]. Previously, the effect of amaranth intervention on the Apos and ox-LDL levels was assessed by Kabiri et al. [32] who compared the effect of 60 days’ intervention with a standard diet, standard diet and cholesterol, standard diet and *Amaranthus caudatus L.* extract, standard diet and amaranth extract with cholesterol, and standard diet with lovastatin and cholesterol in rabbits. The authors reported decreased ox-LDL and ApoB levels and increased ApoA concentrations in animals fed a high cholesterol diet and amaranth extract compared to the group fed a high cholesterol diet only. Importantly, amaranth extract was more effective than lovastatin in decreasing ApoB and ox-LDL levels. On the other hand, we noted that amaranth oil slightly increased ApoB levels compared to the rapeseed oil, consequently the ApoB/A1 ratio, which may indicate that as compared to amaranth oil, rapeseed oil may have more beneficial cardio-protective effect. This may be partly explained by increased TC and LDL-C levels in the amaranth group compared to the rapeseed group as we previously reported [19]. The different effects of amaranth and rapeseed oils on ApoB levels and ApoB/A1 ratio may be also related to their different fatty acid profiles. Rapeseed oil contains MUFA and PUFA and is a good source of sterols [7], whereas amaranth oil contains less MUFA and n-3 PUFA and displays a poorer ratio of UFA to SFA. Amaranth oil also has a lower n-3 to n-6 PUFA ratio but contains a high amount of squalene [29]. Importantly, amaranth oil also contains more palmitic acid [39].

Previously, several studies assessed the effect of amaranth intervention on glucose and insulin metabolism, showing that amaranth grain and oil supplementation in streptozotocin-induced diabetic rats improved glucose levels, increased insulin concentrations and had a beneficial effect on lipid metabolism [28,40]. Besides, Moszak et al. [29] demonstrated that both amaranth and rapeseed oil supplementation for three weeks together with a calorie-restricted diet reduced fasting insulin and decreased insulin resistance measured by HOMA-IR but amaranth oil was significantly more effective in improving fasting glucose levels. Moreover, the beneficial effect of amaranth oil on parameters of carbohydrate metabolism was also found in subjects with type 2 diabetes mellitus [41]. However, our study found no differences between the effect of rapeseed and amaranth oils on glucose and insulin homeostasis parameters. It is possible that the intervention period was too short to detect significant differences between groups. However, in comparison to rapeseed oil, amaranth oil had a slight favorable effect on adiponectin levels. Adiponectin is secreted by adipose tissue, has anti-inflammatory properties and plays an important role in regulating insulin sensitivity and lipid metabolism. Previously, decreased levels of adiponectin were associated with the development of atherosclerosis and insulin resistance [42]; therefore, the observed increase in adiponectin levels in the amaranth group compared to the rapeseed group may be beneficial. However, these results should be confirmed in other randomized trials, as, to our knowledge, this is the first study that assessed the effect of amaranth oil on adiponectin levels. Nevertheless, the effect of amaranth consumption on other adipokines in subjects with diabetes was assessed by Gómez-Cardona et al. [43], showing that leptin, resistin and visfatin levels decreased in normal weight, overweight and obese subjects after three months of consumption of 20 g of amaranth per day.

The study has some strengths and limitations. The main strengths of this study were the cross-over randomized controlled design. Moreover, this is one of the first human studies to compare the effect of amaranth and rapeseed oils on Apos, ox-LDL, adiponectin and TNF-α levels. The major limitation of this trial was the relatively short duration of the intervention period. Moreover, the study was conducted in overweight and obese adult Caucasian population. Therefore, our findings could not be generalized to other populations (e.g., children or adults with normal body weight) and ethnic groups. Besides, we measured only one pro-inflammatory marker (TNF-α) and did not assess oxidative stress parameters. The lack of a separate control group may be considered as another limitation of the study. We also did not assess other factors that could have a potential impact on the obtained results such as alcohol consumption, smoking and physical activity. However, all participants were instructed to maintain their normal physical activity level and eating habits. Furthermore, we did not analyze the composition of oils used in the study. Their composition depends on species and production methods and can vary [44]. Therefore, we could not estimate the exact dose of squalene that the subjects received per day. Moreover, we compared the effect of amaranth oil with rapeseed oil, which has a proven beneficial effect on atherosclerosis markers [45,46]. Therefore, it is possible that the results could be more promising if we compared amaranth oil with another type of oil (e.g., sunflower oil).

## 5. Conclusions

Amaranth oil does not have a greater beneficial effect on atherosclerosis markers than rapeseed oil. However, further studies with a longer intervention period are needed to confirm this effect. Moreover, further studies should also assess the content of squalene in amaranth oil and analyze the dose-response effect.

## Figures and Tables

**Table 1:** Baseline characteristics of the study population.

	Total (*n* = 44)		Arm I (*n* = 23)		Arm II (*n* = 21)		*p*
	Mean ± SD (95%CI)	Median (IQR)	Mean ± SD (95%CI)	Median (IQR)	Mean ± SD (95%CI)	Median (IQR)	
Sex [% of women] ^1^	32 (72.7%)		17 (73.9%)		15 (71.4%)		0.8534
Age [years]	49 ± 10 (46–52)	49 (42–56)	49 ± 9 (45–54)	51 (42–59)	48 ± 11 (43–53)	48 (42–56)	0.5409
Body weight [kg]	87.7 ± 15.1 (83.1–92.3)	89.8 (73.4–100.3)	83.8 ± 16.3 (76.8–90.9)	85.9 (68.8–95.6)	91.9 ± 12.6 (86.2–97.7)	90.2 (85.8–101.4)	0.1074
BMI [kg/m^2^]	31.00 ± 4.61 (29.60–32.40)	30.60 (27.44–33.16)	29.86 ± 4.65 (27.85–31.87)	28.36 (25.73–32.35)	32.25 ± 4.33 (30.27–34.22)	31.04 (30.10–34.52)	0.0668
TNF-α [pg/mL]	4.91 ± 1.19 (4.55–5.27)	4.85 (3.87–5.70)	4.88 ± 1.12 (4.40–5.36)	4.85 (3.87–5.52)	4.94 ± 1.29 (4.35–5.53)	4.91 (3.79–6.07)	0.6382
ox-LDL [ng/mL]	722.6 ± 357.3 (613.9–831.2)	498.3 (428.3–1200.0)	704.3 ± 347.0 (554.2–854.3)	502.8 (420.8–1170.3)	742.6 ± 375.7 (571.6–913.6)	487.4 (446.5–1200.0)	0.8304
ApoE [µg/mL]	101.5 ± 67.1 (81.1–121.9)	82.6 (62.8–120.4)	99.7 ± 68.6 (70.1–129.4)	75.2 (54.8–125.6)	103.5 ± 67.2 (72.9–134.1)	85.6 (65.2–106.0)	0.6217
ApoA1 [g/l]	1.67 ± 0.35 (1.57–1.78)	1.67 (1.43–1.85)	1.64 ± 0.39 (1.48–1.82)	1.58 (1.41–1.90)	1.70 ± 0.31 (1.56–1.84)	1.72 (1.55–1.83)	0.5333
ApoB [g/l]	1.08 ± 0.26 (1.00–1.16)	1.01 (0.95–1.18)	1.10 ± 0.28 (0.98–1.23)	0.99 (0.94–1.25)	1.05 ± 0.23 (0.95–1.16)	1.01 (0.95–1.09)	0.8051
ApoB/A1	0.68 ± 0.23 (0.61–0.75)	0.62 (0.51–0.81)	0.71 ± 0.27 (0.60–0.83)	0.72 (0.49–0.92)	0.64 ± 0.17 (0.56–0.71)	0.58 (0.53–0.75)	0.4521
Glucose [mg/dl]	105 ± 14 (100–109)	101 (97–108)	102 ± 10 (98–107)	102 (96–112)	108 ± 18 (100–116)	100 (98–107)	0.5100
Inulin [µU/mL]	18.1 ± 15.4 (13.4–22.7)	13.4 (9.1–19.3)	13.6 ± 6.4 (10.8–16.3)	12.1 (8.4–19.0)	22.9 ± 20.4 (13.6–32.3)	13.6 (11.2–24.1)	0.1842
HOMA	5.00 ± 5.68 (3.27–6.73)	3.36 (2.36–5.10)	3.46 ± 1.78 (2.68–4.23)	2.91 (2.06–4.36)	6.69 ± 7.75 (3.16–10.22)	3.48 (2.63–5.83)	0.1326
QUICKI	0.53 ± 0.08 (0.51–0.56)	0.53 (0.49–0.58)	0.55 ± 0.07 (0.52–0.58)	0.55 (0.50–0.60)	0.51 ± 0.08 (0.47–0.55)	0.53 (0.47–0.57)	0.1326
Adiponectin [µg/mL]	7.55 ± 4.50 (6.18–8.92)	6.19 (4.56–8.76)	7.89 ± 4.64 (5.89–9.90)	7.85 (4.22–11.61)	7.17 ± 4.43 (5.15–9.19)	5.80 (4.58–7.64)	0.6384

^1^ n (%); Apo–apolipoprotein; BMI–body mass index; HOMA–homeostatic model assessment of insulin resistance; IQR–interquartile range; ox-LDL–oxidized low-density lipoprotein; SD–standard deviation; TNF-α–tumor necrosis factor-alpha; QUICKI–quantitative insulin sensitivity check index; 95% CI–95% of a confidence interval.

**Table 2:** Differences in changes in analyzed parameters between groups.

	Amaranth Oil		Rapeseed Oil		*p*
	Mean ± SD (95%CI)	Median (IQR)	Mean ± SD (95%CI)	Median (IQR)	
Δ TNF-α [pg/mL]	0.03 ± 0.90 (−0.24–0.30)	0.21 (−0.58–0.60)	0.16 ± 1.55 (−0.31–0.63)	0.21 (−0.73–0.54)	0.9071
Δ ox-LDL [ng/mL]	1.2 ± 89.5 (−26.0–28.4)	0.0 (−46.9–31.5)	−7.6 ± 101.7 (−38.5–23.3)	0.0 (−56.5–41.2)	0.7005
Δ ApoE [µg/mL]	1.4 ± 43.6 (−11.9–14.7)	2.6 (−16.6–27.7)	−7.0 ± 45.9 (−20.9–6.9)	−7.6 (−16.2–11.5)	0.3104
Δ ApoA1 [g/l]	0.03 ± 0.22 (−0.04–0.10)	0.02 (−0.11–0.20)	−0.01 ± 0.21 (−0.07–0.06)	0.01 (−0.10–0.11)	0.6526
Δ ApoB [g/l]	0.05 ± 0.20 (−0.01–0.11)	0.07 (0.01–0.14)	−0.03 ± 0.11 (−0.07–0.00)	−0.01 (−0.12–0.03)	0.0004
Δ ApoB/A1	0.01 ± 0.14 (−0.03–0.05)	0.04 (0.00–0.07)	−0.02 ± 0.06 (−0.04–0.00)	−0.01 (−0.06–0.02)	0.0113
Δ glucose [mg/dl]	−2 ± 10 (−5–1)	−3 (−8–5)	−2 ± 15 (−6–3)	−3 (−8–5)	0.4763
Δ inulin [µU/mL]	−1.4 ± 12.6 (−5.2–2.5)	−0.6 (−4.8–1.9)	−2.5 ± 14.7 (−7.0–2.0)	−1.6 (−4.3–0.9)	0.1352
Δ HOMA	−0.58 ± 4.14 (−1.84–0.68)	−0.30 (−1.21–0.51)	−0.74 ± 6.14 (−2.61–1.12)	−0.47 (−1.41–0.20)	0.1446
Δ QUICKI	0.01 ± 0.05 (−0.01–0.02)	0.01 (−0.03–0.05)	0.03 ± 0.06 (0.01–0.04)	0.02 (−0.01–0.06)	0.1758
Δ adiponectin [µg/mL]	0.55 ± 1.10 (0.22–0.89)	0.46 (0.02–1.01)	−0.29 ± 1.50 (−0.75–0.16)	−0.36 (−0.94–0.16)	0.0002

Apo–apolipoprotein; HOMA–homeostatic model assessment of insulin resistance; IQR–interquartile range; ox-LDL–oxidized low-density lipoprotein; SD–standard deviation; TNF-α–tumor necrosis factor-alpha; QUICKI–quantitative insulin sensitivity check index; 95% CI–95% of the confidence interval; Δ–delta/changes (post- minus preintervention values).

## Data Availability

The data presented in this study are available on request from the corresponding author. The data are not publicly available due to the disagreement of the study participants.

## Supplementary Materials

The following are available online at https://www.mdpi.com/article/10.3390/ijerph18168540/s1 (accessed on): Table S1: CONSORT 2010 checklist of information to include when reporting a randomized trial.
